# Residual Fatigue Properties of Asphalt Pavement after Long-Term Field Service

**DOI:** 10.3390/ma11060892

**Published:** 2018-05-25

**Authors:** Peide Cui, Yue Xiao, Mingjing Fang, Zongwu Chen, Mingwei Yi, Mingliang Li

**Affiliations:** 1 State Key Laboratory of Silicate Materials for Architectures, Wuhan University of Technology, Wuhan 430070, China; cuipeide@whut.edu.cn; 2 School of Transportation, Wuhan University of Technology, Wuhan 430070, China; mingjingfang@whut.edu.cn; 3 Faculty of Engineering, China University of Geosciences (Wuhan), Wuhan 430074, China; 4 Research Institute of Highway Ministry of Transport, Beijing 100088, China; cwhymw@gmail.com (M.Y.); li@rioh.cn (M.L.); 5 National Engineering Research Center of Road Maintenance Technologies, Beijing 100095, China

**Keywords:** asphalt pavement, fatigue property, pavement failure, long-term field service

## Abstract

Asphalt pavement is widely used for expressways due to its advantages of flexibility, low cost, and easy maintenance. However, pavement failures, including cracking, raveling, and potholes, will appear after long-term service. This research evaluated the residual fatigue properties of asphalt pavement after long-term field service. Fatigue behavior of specimens with different pavement failure types, traffic load, service time, and layers were collected and characterized. Results indicate that after long-term field service, surface layer has a longer fatigue life under small stress levels, but shorter fatigue life under large stress levels. Longer service time results in greater sensitivity to loading stress, while heavier traffic results in shorter fatigue life. Surface and underneath layers present very close fatigue trend lines in some areas, indicating that the fatigue behavior of asphalt mixture in surface and underneath layers are aged to the same extent after eight to ten years of field service.

## 1. Introduction

Most highways in the world are asphalt pavements. Due to numerous failures of asphalt pavements, which are brought on by heavy vehicles, high volume traffic, and adverse weather, service life of highways is shorter than expected [1,2,3]. Fatigue fracture is one of the most serious failures pavement can undergo. Fatigue fracture is defined as the phenomenon of deterioration of a material (reduction in stiffness and strength, ending in fracture). Fatigue fracture occurs in areas subjected to repeated traffic loading (wheel paths), and may consist of a series of interconnected cracks in early stages of development [4]. In later stages, fatigue fracture develops into many-sided, sharp-angled pieces, with a characteristic alligator pattern [5,6]. Fatigue fractures decrease the structural capacity of pavements. Furthermore, once fatigue fractures propagate through the entire asphalt thickness, water and aggressive agents have a greater opportunity to infiltrate into the unbound layers, which greatly accelerates the deterioration process [7,8,9]. Various laboratory methods were used to simulate the actual traffic environment such that fatigue performance of asphalt pavements could be assessed efficiently and professionally.

To extend the life span of asphalt pavement [10,11], many studies have been conducted to understand the fatigue properties from many aspects, such as influence of asphalt, occurrence of fatigue, and how to prevent expanding of the fatigue fractures. Fatigue tests are divided into the simple flexural tests (two-point, three-point, and four-point bending), uniaxial loading test, and diametral loading test (indirect tensile test) [12]. The indirect tensile (IDT) fatigue test is a convenient and practical method for testing fatigue performance of asphalt pavement. IDT is a part of the recommend standard test methods in European standard EN 12697-24. Roque et al. characterize the crack growth rate of asphalt mixtures by using IDT fatigue tests [13]. Kim et al. built a fatigue model for asphalt pavement, which can predict the fatigue life of asphalt mixtures efficiently [14]. Hafeez et al. investigated the fatigue performance of field-laid asphalt mix specimens [15]. Jiang et al. used semi-circular bending (SCB) strength test and fatigue test to investigate the strength and fatigue properties of asphalt mixtures [16]. It is also well known that the study of asphalt aging through field specimens can offer more meaningful information on the performance of asphalt pavements. Praticò et al. model the dependence of the pavement life-cycle cost on asphalt binder quality, and determine the quantitative relationship between bitumen viscosity and the pay adjustment (PA) for a given class of boundary conditions. They found that asphalt binder viscosity can strongly affect the expected pavement life and the PA, and thus needs to be taken into account in contract and construction management [17]. Elkashef et al. used dynamic shear rheometer (DSR), bending beam rheometer (BBR), and disk compact tension (DCT) tests to evaluate rejuvenated RAP (Reclaimed Asphalt Pavement) binder and mixtures. Considerable improvements in fatigue cracking resistance of rejuvenated RAP binder and fracture energy of rejuvenated RAP mixtures were found [18].

Roads can undergo numerous types of failure, so it is necessary to research the fatigue performance of field samples with different failure types. The main objective of this research is to study the residual fatigue characteristics of field specimens collected from expressways that have served for seven, eight, and ten years. Specimens were cored from locations with different failures, like high-severity transverse cracking, longitudinal cracking, alligator cracking, potholes, and raveling. They were detected by IDT fatigue test. The following research studies residual fatigue behavior of specimens after long-term field service, with consideration of different failure types, daily traffic load, service time, and layer positions.

## 2. Materials and Research Methodologies

### 2.1. Materials

Expressways located in Hebei Province, China, which have multiple failures, were selected in this study based on the pavement service time and traffic volume characteristics. Hebei province is located in East Asia, which has a temperate continental monsoon climate with an annual average temperature of 9–12.6 °C.

Field specimens used in this study were obtained from pavements using a coring machine. Pavements were cored in the places that have various failures, including high-severity transverse cracking, longitudinal cracking, alligator cracking, potholes, and raveling. Field obtained cores should have relatively smooth, parallel surfaces, and conform to the height and diameter requirements for laboratory IDT fatigue tests. In this study, specimens with full pavement depth were sawed and separated from the layer interface, and tested separately. The information for specimens with different failure types is presented in Table 1.

**Figure 1:**
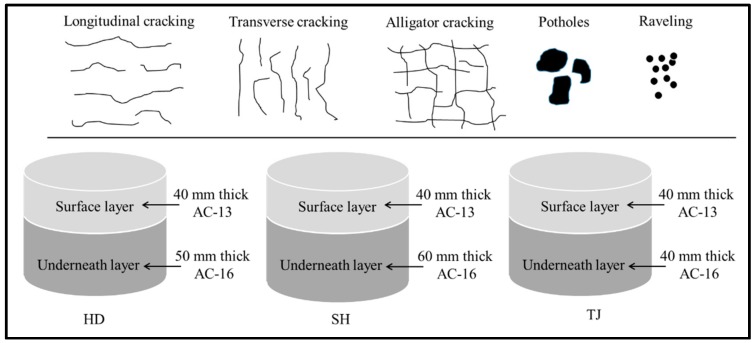
Material information and failure types.

HD expressway’s daily traffic is the smallest of the three highways, and SH expressway’s traffic flow is larger than that of HD. Daily traffic of the TJ expressway is the largest, which is two times higher than that of HD expressway. TJ expressway was constructed and available for service in 1994, and was treated with a new surface layer in 2007. Specimens of the TJ expressway were cored in 2014. SH expressway has been in service since 2006 and specimens were collected from field in 2014. The HD expressway had been in service for more than 10 years before it was cored. Both layers used SBS (Styrene-Butadiene-Styrene) modified asphalt binder. Figure 1 summarizes the detailed information of cores used in this research.

### 2.2. Research Methodologies

#### 2.2.1. Research Program

Firstly, residual fatigue properties of specimens from one expressway were characterized using IDT fatigue test according to failure types to minimize the influence resulting from material and structural variation. Then, specimens’ fatigue characteristics from three expressways were tested and analyzed according to failure types, traffic volume, and service time.

#### 2.2.2. Indirect Tensile Fatigue Test

To get the loading values that were needed in fatigue test, indirect tensile strength (ITS) tests were conducted before fatigue tests. The ITS test was performed at 15 °C with a constant loading rate of 50 mm/min. This loading generates a relatively uniform tensile stress perpendicular to the direction of applied load and along the vertical diametrical plane, which causes the specimen to fail by splitting along the central part of vertical diameter.

**Figure 2:**
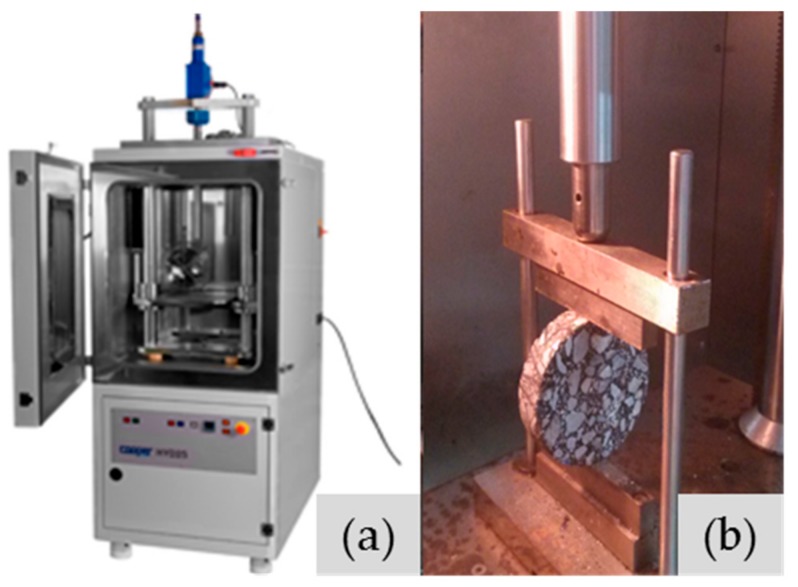
UTM (**a**) and IDT test setup (**b**).

Repetitive IDT fatigue tests were used to measure fatigue performance of asphalt mixtures in accordance with the AASHTO-TP31 standard and they were designed to simulate the tensile forces that are generated in asphalt concrete pavements under traffic loading [19,20,21,22]. Testing machine used is a universal test machine (UTM-25), which was produced by IPC Global in Australia. In the IDT fatigue test, a cylinder-shaped specimen was exposed to repeated compressive loads with a haversine load signal through the vertical diametrical stripe. A picture of the IDT fatigue testing setup is presented in Figure 2.

The environmental chamber used to store specimens was set at 15 °C. Haversine waveform loading was used with 0.1 s loading and 0.9 s resting time. The sample was placed in the chamber at testing temperature for at least 4 h before testing, so that the samples reached the targeted temperature. The tests were performed under stress-controlled conditions according to various stress levels, which calibrate the ratio between applied stress and ITS. There were three specimens tested under the replicated test conditions.

**Figure 3:**
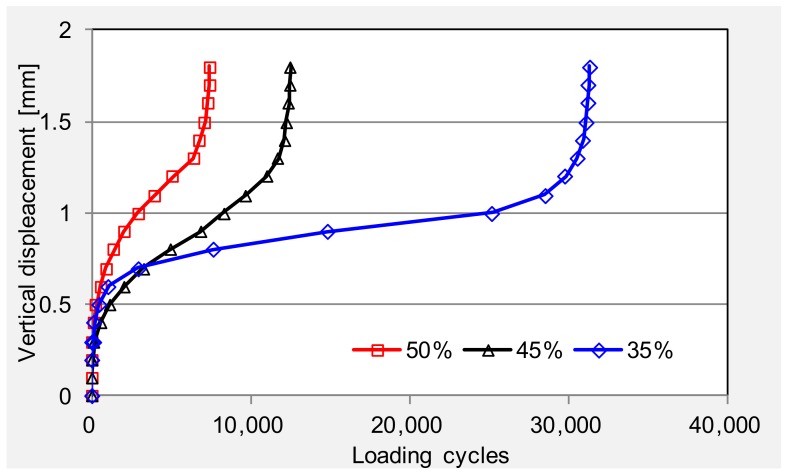
Fatigue curves under three applied stress levels.

Figure 3 compares the fatigue curves of specimens from H-PH-s with three applied stress levels that are 35%, 45%, and 50% of the ITS values. The vertical displacement is going up along with accumulated stress cycles until specimens fail. Three stages can be discovered during the fatigue tests, including crack initialization, crack propagation, and crack failure. The stage of crack propagation is very short when large stress levels are applied, and the crack propagation stage will last longer when smaller stress levels are applied, since asphalt mixture is a kind of viscoelastic material. These results show linear behavior of crack propagation under minor stress and strain levels, whereas beyond stress and strain levels, crack propagation behavior becomes nonlinear because of accumulation of damage, which may be expressed as the development of micro-cracks [23].

There are some different conventional criteria of failure used in the literature. Fatigue failure is normally defined as the moment at which the stiffness has reduced to 50% of its initial value [24]. However, some scholars define fatigue failure as the moment when actual specimen failure was observed [25]. Fatigue failure is also defined using the stress–strain hysteresis loop in each loading cycle of the fatigue test [26,27]. In this research, the fracture fatigue life is determined to be the total number of load applications that caused a complete fracture of the specimen. The vertical deformation detected by UTM’s press head will increase sharply when specimen is completely fractured. So, the number of load applications corresponding to a sharp increase in vertical deformation is the fatigue life in this study.

In fatigue testing, fatigue relationships were represented using a line in the double logarithmic coordinate system, which consisted of *N_f_* and *σ*_0_ in the classical fatigue analysis [28].

(1) $$N_{f}\;=\;K{\left(\frac{1}{\sigma_{0}}\right)}^{n}\;$$

where *N_f_* is the number of cycles to failure, *σ*_0_ is the applied loading level, and *K* and *n* are the coefficients related to the material properties.

#### 2.2.3. Indirect Tensile Resilient Modulus

To provide a reference for fatigue performance analysis and assist the description of the aging degree between the surface and underneath layer of asphalt mixture, specimens were also selected for indirect tensile (IT) resilient modulus tests in this study. IT resilient modulus is one of the basic parameters used to describe the viscoelastic properties of asphalt materials. It is determined by the ratio of stress amplitude to strain amplitude at steady state:

(2) $$E^{*}\;=\frac{4\mathrm{Ph}}{{\pi d}^{2}\Delta L}$$

where E* is resilient modulus (MPa), *P* is load (N), *h* is specimen’s height (mm), *d* is specimen’s diameter (mm), and Δ*L* is resilient deformation.

IT resilient modulus was also found using AASHTO-TP31. Testing machine is UTM-25, which was the same as IT fatigue test. Half-sine intermittent longitudinal loads were applied to the specimens. The system will adjust the applied load according to the target horizontal deformation, and automatically collect the load and displacement data of the last five waveforms. IT resilient modulus is calculated according to the Poisson ratio after obtaining the recoverable vertical deformation of the specimen.

## 3. Results and Discussions

Firstly, specimens from one expressway were studied to minimize the influence resulting from material and structural variation. Then, specimens from three expressways were tested and compared with each other.

### 3.1. Fatigue Properties in One Expressway

Firstly, specimens from HD expressway, which have been in service for more than 10 years, were evaluated. No surface dressing, such as micro-surfacing or slurry seal, has been applied during service life. Therefore, specimens from this expressway can be considered as samples that were directly affected by the environment and traffic.

Indirect tensile strength should be conducted and analyzed before fatigue test. The test was replicated three times and average results are listed in Table 2. Firstly, it is clear that ITS values of surface layer samples are all higher than that of corresponding underneath layer. Secondly, specimens from transverse and longitudinal cracking areas have the same ITS values, regardless of whether surface or underneath layer is measured. Thirdly, ITS tests show that the surface and underneath layers from potholes have the same ITS values, and samples from underneath layer of raveling location present the smallest ITS value.

#### 3.1.1. Cracking

**Figure 4:**
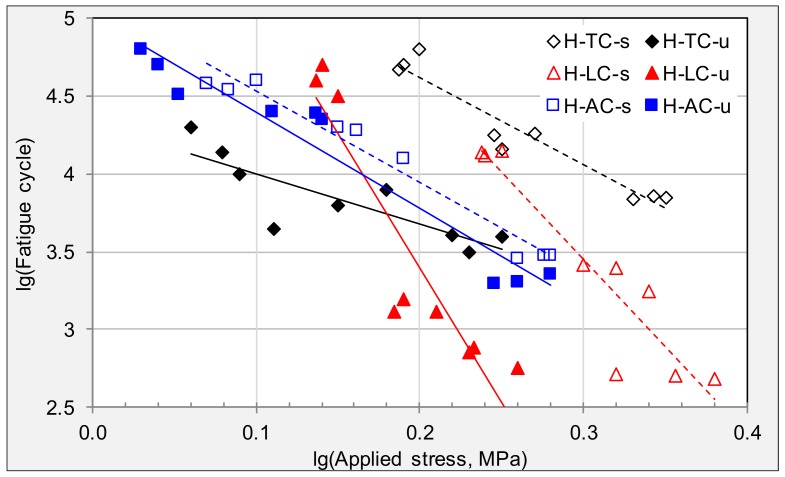
Fatigues trend lines of stress cycles for specimens from cracking areas.

Analysis of fatigue behavior was processed on specimens from cracking areas. Fatigue trend lines and equations for the investigated field specimens from HD are listed in Figure 4 and Table 3, respectively. Simultaneous logarithm on both sides of Equation (1) was conducted before drawing fatigue trend line, therefore log-linear relation can be observed between applied stress and fatigue life. Value of *n* is the linear gradient of fatigue curve, representing the stress sensitivity of the tested specimen, while *K* values stand for level of fatigue life for asphalt pavement.

Samples obtained in different cracking areas vary greatly in their fatigue behavior. Firstly, trend lines state that longitudinal cracking has the largest stress sensitivity, as their slope of fatigue trend lines are the biggest. Secondly, the fatigue life of surface layer is significantly higher than that of corresponding underneath layer for transverse cracking and alligator cracking. The *n* values of the surface layer are similar to the underneath layer in the alligator areas, indicating that surface and underneath layers get aged to the same extent in alligator areas after more than ten years’ field service. Thirdly, *K* and *n* values of the LC area samples are significantly higher than those of other areas. Moreover, the *n* value of the underneath layer is even higher than that of surface layer, indicating that the protective effect of surface layer is lost when the longitudinal cracking occurred.

**Figure 5:**
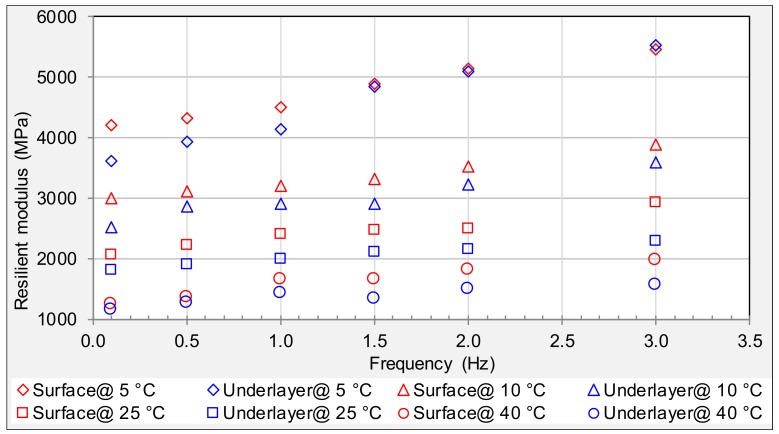
Resilient modulus of specimens from alligator cracking areas.

The IT resilient modulus test was conducted using specimens obtained from alligator cracking areas. Test results are shown in Figure 5, which indicates that the resilient modulus increases with increasing frequency and decreasing experimental temperature. The resilient modulus of specimens from surface and underneath layers is close to each other. This also illustrates that more than 10 years’ field service will result in serious aging in the location of alligator cracking from both surface and underneath layers, which is identical to the fatigue test.

#### 3.1.2. Raveling and Potholes

**Figure 6:**
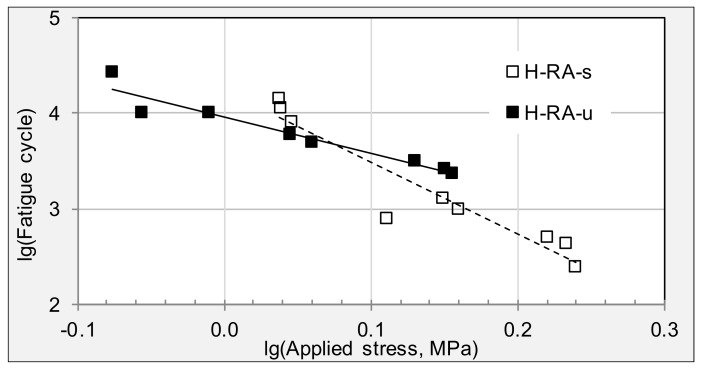
Fatigue trend lines of stress cycles for specimens from raveling areas.

Table 4 summarizes the fatigue equations and parameters for the raveling and pothole field specimens from HD expressways. Compared to specimens from cracking areas, raveling areas have the smallest *K* value, which means the worst fatigue life. Figure 6 shows the fatigue trend lines of specimens from raveling areas. Specimens from surface layer are more sensitive to the applied loading stress compared with underneath layer. The fatigue trend lines between surface and underneath layers are overlapping with each other. These results illustrate that ten years of service has resulted in brittle pavement surface. Cracks and other failures will occur when additional traffic loading is applied.

**Figure 7:**
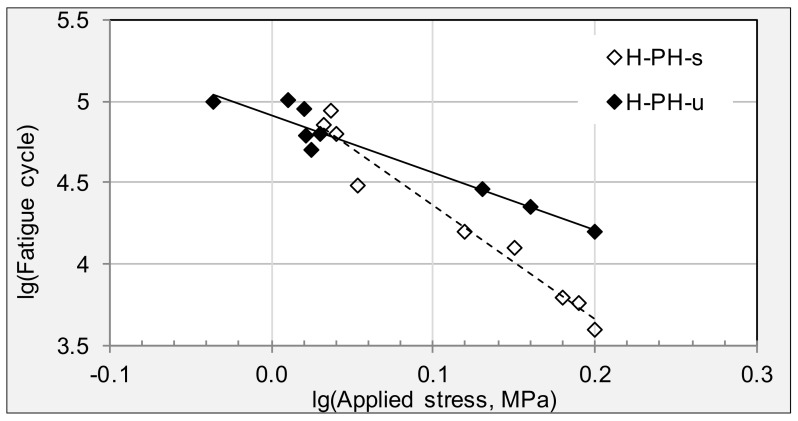
Fatigues trend lines of stress-cycles for specimens from pothole areas.

Figure 7 compares the fatigue trend lines of specimens from potholes. Firstly, similar to results from cracking and raveling areas, surface layer from potholes is more sensitive to the applied loading stress than that of underneath layer. Secondly, specimens from pothole areas have higher fatigue life than that of specimens from raveling areas, regardless of whether surface or underneath layer is examined.

Fatigue results in this section show that surface layer has higher fatigue life under smaller stress levels, but shorter fatigue life under bigger stress levels. Asphalt binder will become hard and brittle after long-term service, leading to higher ITS values and extended fatigue life at lesser stress levels, but severely short fatigue life at larger stress levels. This signifies that after long-term service, the ability of pavement to resist low stress levels increases and to resist high stress levels drops significantly, especially when it comes to longitudinal cracking areas with the largest stress sensitivity. When a small crack appears, the crack will propagate rapidly, resulting in pavement failure.

### 3.2. Fatigue Properties in Different Expressway

#### 3.2.1. Cracking Area

Specimens from SH and TJ expressway cracking areas were tested for comparison. Table 5 presents ITS values found using fatigue tests. Like the results in Table 2, ITS values for the surface layer is higher than that of underneath layer for longitudinal cracking areas, showing that the surface layer from SH expressway has undergone severe aging and hence resulted in hard and brittle composition.

**Figure 8:**
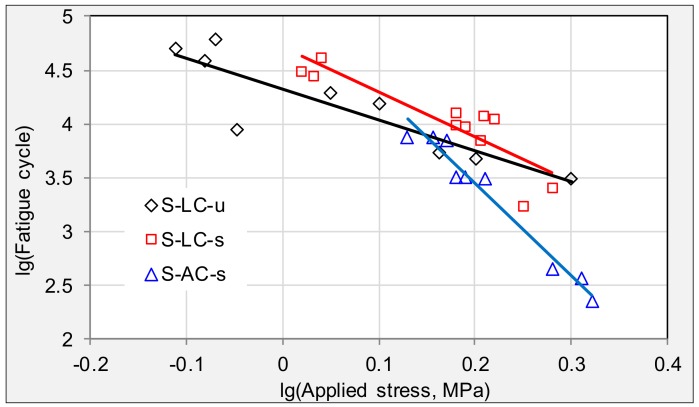
Fatigue trend lines for specimens from cracking areas in SH expressway.

Figure 8 compares the fatigue trend lines of specimens from SH expressway. Fatigue trend lines of surface and underneath layers are quite close, indicating homogenous ageing index had been achieved after eight years’ field service. Specimens from alligator cracking areas present the worst fatigue property, of which *n* value is more than twice that of the longitudinal cracking areas.

**Figure 9:**
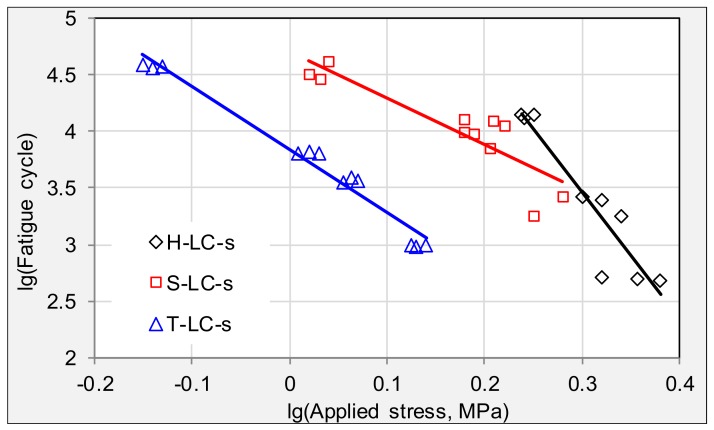
Comparison of fatigue trend lines between varying expressways with longitudinal cracking.

**Figure 10:**
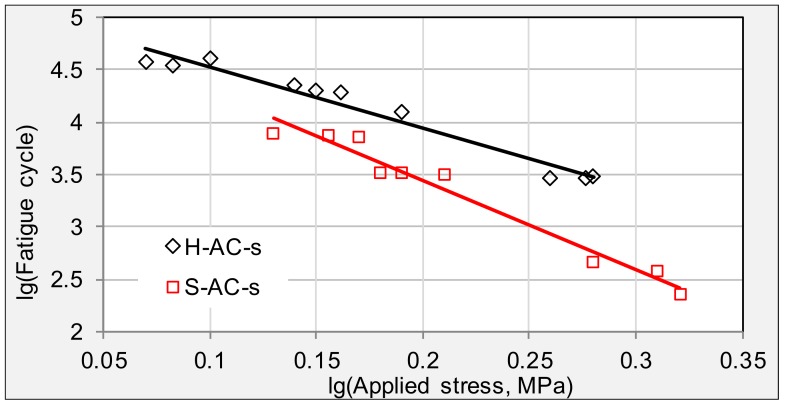
Comparison of fatigue trend lines between varying expressways with alligator cracking.

Figure 9 compares the fatigue behaviors of specimens from longitudinal cracking areas in the HD, SH, and TJ expressways. Figure 10 compares the fatigue results of samples from alligator cracking in HD and SH expressways. The TJ expressway, which has the largest daily traffic, shows the smallest fatigue life, while HD expressway, which has a longer service time than SH and TJ, has the largest stress sensitivity to loading stress. Therefore, severe ageing can be expected on HD specimens, resulting in hard asphalt binder with significantly reduced viscoelasticity. TJ carried the busiest daily traffic with large amount of heavy trucks. Therefore, lowest fatigue life is found in Figure 9 for the TJ expressway. This relationship between traffic load and fatigue performance can also be seen in Figure 10. The traffic load of the SH expressway is higher than that of the HD expressway, which results in the samples from SH having higher stress sensitivity than HD expressway and lower fatigue life than HD expressway. Based on the previous analysis, rules can be summarized stating that heavier daily traffic results in shorter fatigue life, and longer service time results in more sensitivity to loading stress.

#### 3.2.2. Raveling Area

Specimens from raveling areas in SH expressway were studied as well. The ITS values between the surface layer and underneath layer are 3.39 MPa and 3.42 MPa for S-RA-s and S-RA-u, respectively. Once again, the ITS values for two layers is the same, indicating that long field-aging has resulted in severe aging that went through into the second layer.

**Figure 11:**
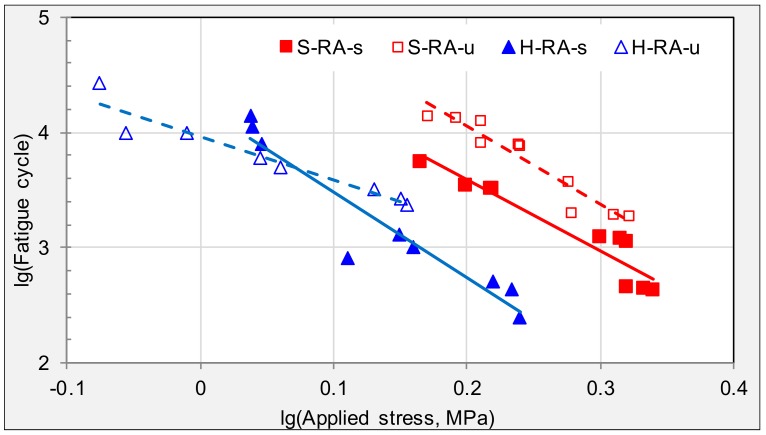
Comparison of fatigue trend lines between varying expressways with raveling.

The fatigue trend lines of specimens from raveling areas in the SH and HD expressways are compared in Figure 11. In the SH expressway, specimens from the underneath and surface layers have similar *n* values. However, in the HD expressway, specimens from the surface layer were more sensitive to applied stresses.

#### 3.2.3. Fatigue Characteristics

Fatigue equations and parameters of the TJ and SH expressways are shown in Table 6. According to Table 3, Table 4 and Table 6, five groups of specimens from surface layer are found to have higher *n* values than the specimens from corresponding underneath layer, except groups H-LC and H-PH. This means that after long-term field service, the surface layer will become severely aged, and hence result in greater sensitivity to the traffic loading. Therefore, failure will be promoted when heavy traffic is applied onto such aged asphalt pavement. Nevertheless, other than the extremely high stress sensitivity in the longitudinal cracking areas of the HD expressway, no obvious relationship was found between the fatigue performance of field samples and the failure types.

The *R*^2^ represents the coefficient of determination for the fatigue curve. The minimum *R*^2^ value is 0.71, which was found for H-TC-u specimen. Such high *R*^2^ values indicate that the analytical methods for fatigue behavior used in this study are largely repeatable. Furthermore, the average *R*^2^ value for HD expressway, which is 0.902, is higher than for the SH expressway, with a value of 0.882. SH expressway is subject to much more daily traffic than that of HD expressway, with two times as many heavy trucks using the HD expressway. Such heavy traffic loading would definitely introduce non-uniform effect on the pavement structure, hence resulting in lower repeatability for the fatigue test.

## 4. Conclusions

The residual fatigue properties of specimens from three long-term aged expressways were studied, according to field failure types, different traffic load, and service life. Specimens from one expressway with 10 years of service history were evaluated first, and then compared with specimens from two other expressways. Based on the research results, the following conclusions can be drawn.

(1) Residual fatigue results show that there is no clear correlation between fatigue properties and pavement failure modes, since potholes and raveling are not simply due to repeated traffic loading. Potholes and raveling are the result of moisture damage phenomenon, along with bitumen quality and adhesion between bitumen and aggregate. Furthermore, the mechanisms for longitudinal cracking and transverse cracking are quite different to what causes alligator cracking, since more than fatigue loading is involved.
(2) The minimum *R*^2^ value for fatigue trend lines is 0.71, illustrating that the fatigue analysis with field specimens in this research has acceptable repeatability. Most specimens from surface layer perform with higher *n* values than the specimens from corresponding underneath layer. Surface layers have a higher fatigue life under small stress levels, but shorter fatigue life under large stress levels, indicating that the materials have been severely aged and the elastic behavior of asphalt mixture has been reduced.
(3) ITS values of surface layer samples are all higher than that of corresponding samples of their underneath layer. Specimens from transverse and longitudinal cracking areas, and their associated surface and underneath layers, have similar ITS values.
(4) In the alligator and longitudinal cracking areas of long-term field service expressways, surface and underneath layers present very close fatigue trend lines, indicating that the fatigue behavior of asphalt mixture in surface and underneath layers are aged to the same extent after ten years or eight years’ field service.
(5) The fatigue performance differs for different expressways. Expressways that carried the busiest daily traffic with large amount of heavy trucks show the lowest fatigue life. While expressway that has the longest service time has the most sensitivity to loading stress. These rules can be summarized by stating that heavier daily traffic results in shorter fatigue life, and longer service time results in more sensitivity to loading stress.

## Figures and Tables

**Table 1:** Information of used cylindrical field specimens.

Expressway No.	Failures	Specimens No.	Field Information
HD expressway	Transversal cracking (TC)	HD-TC-s and -u	Built in 2003; Cored in 2013
	Longitudinal cracking (LC)	HD-LC-s and -u	
	Alligator cracking (AC)	HD-AC-s and -u	
	Potholes (PH)	HD-PH-s and -u	
	Raveling (RA)	HD-RA-s and -u	
SH expressway	Longitudinal cracking (LC)	SH-LC-s and -u	Built in 2006; Cored in 2014
	Alligator cracking (AC)	SH-AC-s	
	Raveling (RA)	SH-RA-s and -u	
TJ expressway	Longitudinal cracking (LC)	TJ-LC-s	Built in 2007; Cored in 2014

**Note.** ‘‘-s” and ‘‘-u” stand for specimens from surface and underneath layer, respectively.

**Table 2:** Indirect tensile strength of asphalt sample from HD expressway.

Indirect Tensile Strength (MPa)	H-TC	H-LC	H-AC	H-RA	H-PH
Surface layer	4.41	4.64	3.32	3.14	2.82
Underneath layer	3.33	3.56	2.76	2.64	2.97

**Table 3:** Fatigue equations of cracking areas in HD expressway.

Specimen No.	Fatigue Equation	Fatigue Parameters		*R* ^2^
		*K*	*n*	
H-TC-s	$N_{f}\;=\;5.58\;\times \;10^{5}{\left(\frac{1}{\sigma_{0}}\right)}^{5.6307}$	$5.58\;\times \;10^{5}$	5.631	0.94
H-TC-u	$N_{f}\;=\;2.12\;\times \;10^{4}{\left(\frac{1}{\sigma_{0}}\right)}^{3.2284}$	$2.12\;\times \;10^{4}$	3.228	0.71
H-LC-s	$N_{f}\;=\;6.33\;\times \;10^{6}{\left(\frac{1}{\sigma_{0}}\right)}^{11.173}$	$6.33\;\times \;10^{6}$	11.173	0.87
H-LC-u	$N_{f}\;=\;8.78\;\times \;10^{6}{\left(\frac{1}{\sigma_{0}}\right)}^{18.061}$	$8.78\;\times \;10^{6}$	18.061	0.84
H-AC-s	$N_{f}\;=\;2.16\;\times \;10^{5}{\left(\frac{1}{\sigma_{0}}\right)}^{6.7652}$	$2.16\;\times \;10^{5}$	6.765	0.96
H-AC-u	$N_{f}\;=\;1.02\;\times \;10^{5}{\left(\frac{1}{\sigma_{0}}\right)}^{6.1724}$	$1.02\;\times \;10^{5}$	6.172	0.94

**Table 4:** Fatigue equations of raveling and pothole areas in HD expressway.

Specimen No.	Fatigue Equation	Fatigue Parameters		*R* ^2^
		*K*	*n*	
H-RA-s	$N_{f}\;=\;3.48\;\times \;10^{4}{\left(\frac{1}{\sigma_{0}}\right)}^{8.1483}$	$3.48\;\times \;10^{4}$	8.148	0.92
H-RA-u	$N_{f}\;=\;9.10\;\times \;10^{3}{\left(\frac{1}{\sigma_{0}}\right)}^{3.8938}$	$9.10\;\times \;10^{3}$	3.894	0.89
H-PH-s	$N_{f}\;=\;8.32\;\times \;10^{4}{\left(\frac{1}{\sigma_{0}}\right)}^{3.5627}$	$8.32\;\times \;10^{4}$	3.563	0.93
H-PH-u	$N_{f}\;=\;8.90\;\times \;10^{4}{\left(\frac{1}{\sigma_{0}}\right)}^{6.3209}$	$8.90\;\times \;10^{4}$	6.321	0.92

**Table 5:** Indirect tensile strength of asphalt samples from SH and TJ expressways.

Indirect Tensile Strength (MPa)	S-LC	S-AC	T-LC
Surface layer	3.34	3.31	2.29
Underneath layer	2.01	--	--

**Table 6:** Fatigue equations of specimens in SH and TJ expressways.

Specimen No.	Fatigue Equation	Fatigue Parameters		*R* ^2^
		*K*	*n*	
S-LC-s	$N_{f}\;=\;4.06\;\times \;10^{4}{\left(\frac{1}{\sigma_{0}}\right)}^{3.0009}$	$4\;\times \;10^{4}$	3.001	0.88
S-LC-u	$N_{f}\;=\;2.13\;\times \;10^{4}{\left(\frac{1}{\sigma_{0}}\right)}^{2.9025}$	$2.13\;\times \;10^{4}$	2.903	0.78
S-AC-s	$N_{f}\;=\;1.27\;\times \;10^{5}{\left(\frac{1}{\sigma_{0}}\right)}^{8.2614}$	$1.27\;\times \;10^{5}$	8.261	0.95
S-RA-s	$N_{f}\;=\;2.05\;\times \;10^{5}{\left(\frac{1}{\sigma_{0}}\right)}^{6.725}$	$2.50\;\times \;10^{5}$	6.725	0.91
S-RA-u	$N_{f}\;=\;6.34\;\times \;10^{4}{\left(\frac{1}{\sigma_{0}}\right)}^{6.0854}$	$6.34\;\times \;10^{4}$	6.085	0.90
T-LC-s	$N_{f\;}\;=\;3.86\;\times \;10^{4}{\left(\frac{1}{\sigma_{0}}\right)}^{3.3757}$	$3.86\;\times \;10^{4}$	3.376	0.92
